# Integration of national cancer registry program with Ayushman Bharat Digital Mission in India: A necessity or an option

**DOI:** 10.1016/j.puhip.2022.100263

**Published:** 2022-04-26

**Authors:** Shubharanjan Jena, Venkatarao Epari, Krushna Chandra Sahoo

**Affiliations:** a Department of Community Medicine, Institute of Medical & Sum Hospital, Siksha ‘O’ Anusandhan, Bhubaneswar, Odisha, India, 751003; b Public Health Specialist, ICMR-Regional Medical Research Centre, Bhubaneswar, Odisha, India, 751023

## 1 Introduction

The cancer registry is one of the essential components and plays a critical role in the cancer control program in a country. The National Cancer Registry Program (NCRP), which began in December 1981 under the auspices of the Indian Council of Medical Research (ICMR), now boasts with 236 hospital-based cancer registries (HBCR) and 36 population-based cancer registries (PBCR) [1]. According to the World Cancer Report 2020, the cancer burden is increasing unequally impacting LMI (low- and middle-income) countries. The report argues that cancer is either first or the second leading cause of premature death among people aged 30–69 years, with India at the 2nd rank. An estimated 7.8 lakh cancer deaths and 1.16 million new cancer cases have been reported from India's population of 1.35 billion. Further, during the life time 1 in 15 Indians have a risk of death, while 1 in 10 Indians will develop new cancers. The World Health Organization in 2017, advocated national governments to fast-track actions to achieve the targets as per the global action plan for the prevention and control of NCDs and the 2030 UN Agenda to reduce premature mortality from cancer [2]. Thus Population-based cancer registries (PBCRs) are the benchmark for determining the true incidence of cancer in any given population. Where as in India the population based cancer registry coverage is only 10% and in rural, it covers only 0.1% of the population [3].

## 2 Landscape of Ayushman Bharat Digital Mission in India

On September 27, 2021, the Honorable Prime Minister of India inaugurated the Ayushman Bharat Digital Mission (ABDM). It was announced in the previous year, during the 74th Independence Day celebration as National Digital Health Mission. Initially, it is being piloted in six union territories, namely Puducherry, Chandigarh, Ladakh, Lakshadweep, Dadra and Nagar Haveli and Daman and Diu and Andaman and Nicobar Islands [4]. In articulating India's National Cancer Registry Program, we have attempted to assess the mission's critical objectives.

Under this mission, each Indian will receive a 16-digit unique health ID generated using Aadhar and mobile numbers. It intends to contain person's health records, including medical tests, diseases, and previous prescriptions, confidentially. The primary purpose is to efficiently support to reduce the out of pocket expenditure and ensure the realization of universal health coverage. The mission's goal is to create a digital health system that will boost India's health care system's efficiency, effectiveness, and transparency [4].

To reduce the economic burden of six groups of common and high-cost diseases, including cancer, the government of India initiated the Ayushman Bharat scheme in 2018. However, since health is a state subject, Ayushman Bharat has not yet been implemented in a few of the states like Telangana, Delhi, West Bengal and Odisha. States like West Bengal and Odisha have their health insurance policy called Swasth Sathi and Biju Swasthya Kalyan Yojana. Therefore, implementing the ABDM across the country is a big challenge [5,6].

## 3 Importance of digitalization in cancer registry

Digitalization plays a vital role in the proper functioning of cancer registries. It results in complete treatment information and data entry, accessing patient data, completeness, patient tracking for follow-up, data retrieval, and increased organizational work output [7]. In China, a study found that modern digitalization technology plays a significant role in managing patient data collection and clinical data in cancer registries. The use of contemporary technology in cancer registries enables healthcare providers to track and record patients' clinical progress, treatment history, easy-to-use tools, and biomarker data. Meanwhile, cancer registry digitization can benefit both researchers and clinical practice [8].

## 4 Necessity of integration

As per the Report of NCRP 2020, cancer registries need to be linked to several other databases (Ayushman Bharat, other insurance schemes, mortality databases, Health Management Information System) both at national and local levels for a seamless improvement of cancer statistics [9]. Though the cancer registry has started in India since 1981, it has overlooked a few large and less developed states. Further, as the death reporting system is poor in India, without mention of the cause of mortality, it needs improvement for the robust tracking of cancer burden [10]. The ‘ABDM’ describes the registry for health professionals and health facilities. However, cancer registries being the backbone of cancer control program the national health authority did not include the cancer registry program in the ABDM mission [4]. As digitalization play a crucial role for the functioning of cancer registry and still in India the registry coverage is very low [3,7]. Thus we believe that the national cancer registry programme should be integrated with Ayushman Bharat Digital Mission (ABDM) to strengthen the process of cancer registration across the country. There are still many unmet needs and a patchwork of public health infrastructure in countries like India; this initiative will aid in the country's pursuit of universal health coverage.

## Ethics statement

This paper is a commentary, so it did not need ethical consideration.

## Funding sources

This research did not receive any specific grant from funding agencies in the public, commercial, or not-for-profit sectors.

## Declaration of competing interest

The authors declare that they have no known competing financial interests or personal relationships that could have appeared to influence the work reported in this paper.
